# Preliminary Retrospective Analysis of Daily Tomotherapy Output Constancy Checks Using Statistical Process Control

**DOI:** 10.1371/journal.pone.0147936

**Published:** 2016-02-05

**Authors:** Emilio Mezzenga, Vincenzo D’Errico, Anna Sarnelli, Lidia Strigari, Enrico Menghi, Francesco Marcocci, David Bianchini, Marcello Benassi

**Affiliations:** 1 Medical Physics Department, Istituto Scientifico Romagnolo per lo Studio e la Cura dei Tumori (IRST) IRCCS, Meldola, FC, Italy; 2 Laboratory of Medical Physics and Expert Systems, Regina Elena National Cancer Institute, Rome, Italy; Cardiff University, UNITED KINGDOM

## Abstract

The purpose of this study was to retrospectively evaluate the results from a Helical TomoTherapy Hi-Art treatment system relating to quality controls based on daily static and dynamic output checks using statistical process control methods. Individual value X-charts, exponentially weighted moving average charts, and process capability and acceptability indices were used to monitor the treatment system performance. Daily output values measured from January 2014 to January 2015 were considered. The results obtained showed that, although the process was in control, there was an out-of-control situation in the principal maintenance intervention for the treatment system. In particular, process capability indices showed a decreasing percentage of points in control which was, however, acceptable according to AAPM TG148 guidelines. Our findings underline the importance of restricting the acceptable range of daily output checks and suggest a future line of investigation for a detailed process control of daily output checks for the Helical TomoTherapy Hi-Art treatment system.

## Introduction

Quality control (QC) processes relating to radiotherapy treatment systems are the planned, systematic actions taken to verify whether a particular machine function is within specific tolerance levels, ensuring the correct operation and constancy function of the system itself. When performing a machine QC, the operator measures the process quality performance by comparing this with existing standards and adopts actions necessary to maintain conformance with standards. This includes setting reference specifications for the treatment system function, measuring its performance, comparing measured data with specifications and, when necessary, using corrective actions to meet specifications. The non-trivial task for the operator during a QC process is that of separating random errors from systematic errors in data collection to avoid re-engineering the process and to ensure that only random errors are present in the data. The statistical process control (SPC) method [1] is the application of statistical and graphical techniques to a process of interest; its aim is to control and improve the QC process by identifying random and systematic errors in a stream of sequential data.

The key concept of SPC techniques is the control chart, *i*.*e*. a plot of measured characteristics of a process showing how these vary over time. A center line (CL) superimposed on the plotted data points corresponds to the average of the process and is used as reference for data point dispersion. An upper and lower control line called upper control limit (UCL) and lower control limit (LCL), characterize the range of the process. A data point falling inside this UCL-LCL range represents a situation of a process in control whose data are only affected by random errors. Points falling outside the UCL-LCL range indicate a process out of control, *i*.*e*. systematic errors present in the measurements.

In state-of-the-art clinical radiotherapy, several publications [2–5] have underlined the importance and usefulness of control charts. They have been used to study the variability in radiotherapy planning for breast cancer patients after mastectomy [2]; to investigate the variability in tumor volumes delineated by different physicians [3]; and to identify systematic errors in the measurements of daily Linac beam characteristics [4, 5] and patient-specific radiotherapy treatments [6–8]. All of these studies have shown that the use of SPC tools allows for a consistently high level of performance in the areas examined, identifying trends and drifts in the data due to specific causes.

Although SPC analysis has been successfully applied to clinical external beam radiotherapy, to the best of the authors’ knowledge it has never been applied to Hi-Art quality controls. In the present work, we used the tools and methods of the SPC, in particular, individual value X-charts and exponentially weighted moving average (EWMA) charts to carry out a retrospective analysis of daily static and dynamic output checks performed on a Helical Tomotherapy (HT) Hi-Art treatment system (Accuray, Inc., Madison, WI, USA) present in our institute. We evaluated the capability of these process control tools applied to the machine outputs to prevent system failure. The aim of our study was to assess the potential for reducing the acceptance range proposed in AAPM TG148 and to implement SPC methods into a clinical context for daily TomoTherapy system delivery control.

## Materials and Methods

### Static and dynamic output

From January 2014 to January 2015, a total of 305 daily static and dynamic output checks of the HT treatment system were measured. Although the AAPM TG 148 [9] suggests that “If the static output is monitored on a daily basis, the rotational output should be monitored on a weekly basis and vice versa”, for the purpose of our study we decided to monitor both outputs to prevent their drifting apart. Moreover, having installed the dose control system (DCS) in our HT system to minimize output variation, the choice of monitoring both outputs was a test bench for the DCS. Both outputs were monitored using a cylindrical phantom supplied by the manufacturer (TomoPhantom, Accuray, Inc., Madison, WI, USA), together with an Exradin A1SL 0.05cc ionization chamber (Standard Imaging Inc., Madison, WI, USA) inserted into it. The ionization chamber was connected to an eight-channel electrometer (TomoElectrometer, Standard Imaging Inc., Madison, WI, USA), and all measurements were corrected for pressure and temperature. For both checks, the phantom was positioned onto the HT treatment couch with the ionization chamber inserts disposed along the sagittal direction (perpendicular to the longitudinal axis of the treatment couch) and the ionization chamber inserted 0.5 cm below the center of the phantom.

**Fig 1:**
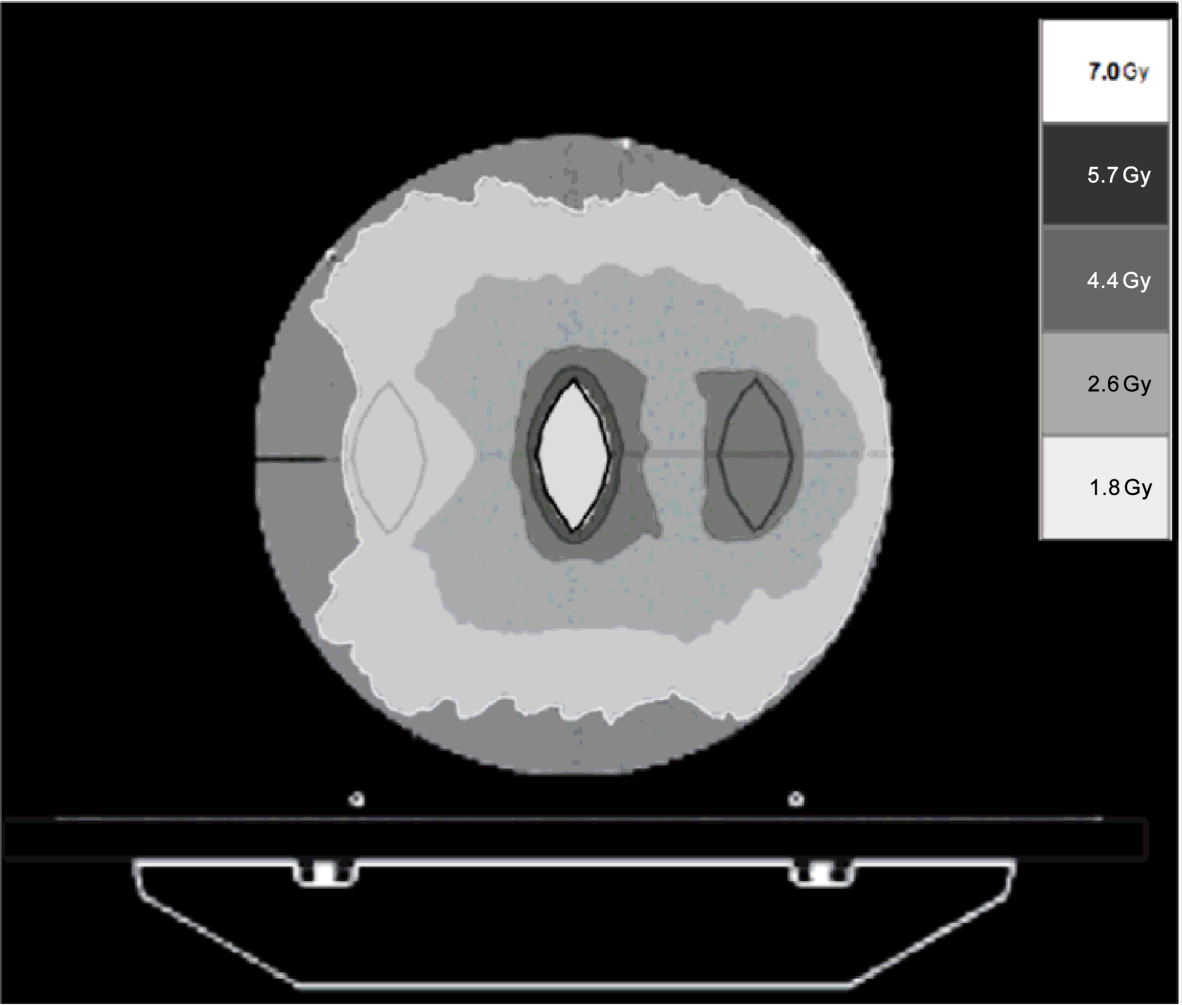
Dose distribution for IMRT treatment plan optimized using the HT TPS on the manufacturer-supplied cylindrical phantom. The reported legend in the figure shows the dose distribution considered in this plan.

As the only recommendations [9] for checking both outputs are a) a stationary gantry and a treatment field delivered for a specific time for the static output check, and b) a rotational procedure generated in the HT treatment planning system (TPS) that mimics patient treatment for the dynamic check, the static output check was performed by delivering a static field size of 5x40 cm^2^ for one minute to the phantom, while a dedicated intensity-modulated radiation therapy (IMRT) plan was optimized on the same phantom using HT TPS (TomoH^TM^, v 5.0.2.5, Accuray Inc.) to check the dynamic output in a clinical setting. The plan consisted of three equal cylindrical targets of semi-elliptical section located inside the phantom and treated with a uniform dose for each target (Fig 1). For both static and dynamic plans, output reference values were established after an acceptance test procedure of our HT system. Subsequently, static and dynamic output measurements were considered acceptable only if their value was within ±3% [9] of the reference value. The ionization chamber reading was considered for both outputs. As the present work was a retrospective observational study of daily HT output checks, ethical committee approval was not necessary.

### Statistical process control

The SPC [1] technique was used to evaluate the process quality of our HT system. Data points were plotted with a UCL, CL and LCL to compare data dispersion values with the range defined by the control limits, and to distinguish between random and systematic errors of data variations. For the former, error reduction requires actions to be taken on the constraints of the process (UCL, CL and LCL) [10–14], whereas the latter requires investigation to find the causes and, where appropriate, action to be taken to eliminate them. This is achieved using process control rules, *i*.*e*. metrics that permit us to interpret control charts and monitor process quality. In our preliminary study of SPC applied to HT static and dynamic output checks, we adopted the general rule that a data point appearing outside the control limits renders the process out of control. This was chosen to avoid too many potential false alarms. A number of authors suggest using control charts [15–17] as these can help the operator to detect either large changes or gradual drifts in the process under investigation. We used individual value X-charts to detect individual value failure and exponentially weighted moving range (EWMA) charts to investigate process drifts. Whilst the X-chart only considers single data points (*i*.*e*. it plots data variation over time), the EWMA chart also uses previous values (*i*.*e*. it is a time-weighted control chart). Given that all measurements in the considered dataset must be independent when using control charts, we only considered daily checks performed with the same ionization chamber. Thus, the 305 starting data points were reduced to 281. Moreover, before starting the HT output check, each ionization chamber to be used in this context was compared with a calibrated reference chamber to evaluate differences in readings. For the X-chart, the first 20 output data points [1] were used to evaluate the control limits (for both static and dynamic output checks) calculated as:

$$UCL=\bar{X}+\frac{\bar{mR}}{d_{2}\sqrt{n}}$$ (1)

$$CL=\bar{X}$$ (2)

$$LCL=\bar{X}-3\frac{\bar{mR}}{d_{2}\sqrt{n}}$$ (3)

where $\bar{X}$ is the average of the dataset considered, $\bar{mR}$ is the average moving range (*i*.*e*. the average of the absolute values of the difference between two consecutive data points ($mR_{i}=\left|x_{i}-x_{i-1}\right|$), and d_2_ is a bias correction constant depending on the subgroup size n. In our case being n = 1, d_2_ = 1.128 [1].

For the EWMA charts, the UCL and LCL were calculated as [18]:

$$UCL=\bar{X}+L\cdot s\sqrt{\frac{l}{2-l}\left[1-{\left(1-l\right)}^{2i}\right]}$$ (4)

$$LCL=\bar{X}-L\cdot s\sqrt{\frac{l}{2-l}\left[1-{\left(1-l\right)}^{2i}\right]}$$ (5)

while CL was defined as in Eq 2). Here, s is the standard deviation of the considered data set, i the sample time point (i = 1,2,..,n), L the width of the control limit, and l the weighting factor (l ⊰] 0;1]) [1]. For large values of i, Eqs 4 and 5 tend to:

$$UCL=\bar{X}+L\cdot s\sqrt{\frac{l}{2-l}}$$ (6)

$$LCL=\bar{X}-L\cdot s\sqrt{\frac{l}{2-l}}$$ (7)

Each time point reported in the EWMA plot was calculated by means of the exponential smoothing equation [17]:

$$S_{i}=\sum_{j=0}^{i-1}l{\left(1-l\right)}^{j}x_{i-j}+{\left(1-l\right)}^{i}\;S_{0}$$ (8)

where *x*_*i-j*_ is the measured data point at sample time i-j (i = 1,2,..,n), and *S*_*0*_ is the starting point of the EWMA chart, generally (as in this study) set equal to the CL of the dataset considered. Given that the l parameter is the rate at which older data enter into the calculation of the EWMA statistic, a small value of l gives more weight to older data and less to new data. We used l = 0.3, and L = 3, as suggested by Čisar et al. [18].

### Data distribution test

X-charts were used to provide process stability, detect out of control situations, and estimate the parameters describing the behavior of the process. The EWMA chart was used as the second phase of data monitoring, with an assumed probability data distribution appropriate to the process.

The null hypothesis (H_0_) is that the measured data points are normally distributed with a chosen alpha risk (the risk of rejecting H_0_ even though it is true) set at 0.01. The Anderson-Darling (AD) test was used to analyze the normal distribution of the dataset considered according to [19]:

$$W_{n}^{2}=-N-\frac{1}{N}\sum_{i}\left(2i-1\right)\{\text{log}\left(F^{*}\left(x_{i}\right)+\text{log}\left(1-F^{*}\left(x_{n+1-i}\right)\right)\right)\}$$ (9)

where F*(x_i_) is the cumulative distribution function of the normal distribution, x_i_ (i = 1,2,..,N) are the ordered collected data (over time), and N is the sample size. The alpha value relies on the assumption that data are within process control limits at 3 standard deviations (*i*.*e*. $3\frac{\bar{mR}}{d_{2}}$). The Eq 9 was modified on the basis of the sample size, as follows [19]:

$$W_{n}^{2}=W_{n}^{2}\left(1+\frac{0.75}{N}+\frac{2.25}{N^{2}}\right)$$ (10)

If the $W_{N}^{2^{*}}$ is less than the alpha risk, the data are normally distributed.

### Process analysis

The process indices C_p_ and C_pk_ were used to quantify the process behavior. In particular, the process capability (C_p_, or capability ratio) was used to compare variations in process data with respect to upper and lower specification limits to quantify the process data dispersion, as follows [20]:

$$C_{p}=\frac{USL-LSL}{6\cdot s}$$ (11)

where USL and LSL are the upper and lower specification limits, respectively (set at ±3.0% [9]), and s is the experimental standard deviation of the data distribution. A C_p_ value of 1.0 indicates that a process is within the specification limits or that the data spread is equal to the specification limit width.

The process acceptability (C_pk_, or acceptability ratio) shows how close the process centre is to the specification limit and is calculated as follows [20]:

$$C_{pk}=\text{min}\left(\frac{USL-\bar{x}}{3\cdot s};\frac{\bar{x}-LSL}{3\cdot s}\right)$$ (12)

$\bar{x}$ being the average process value, *i*.*e*. CL. In Eq 12 it is assumed that the target of the process is equal to 0.0% (measured output checks in complete agreement with the reference values). If the process is centered on the target of the process (output reference values), the capability and acceptability ratio are equal. A higher capability than acceptability ratio means that the process is not centered.

## Results and Discussion

**Fig 2:**
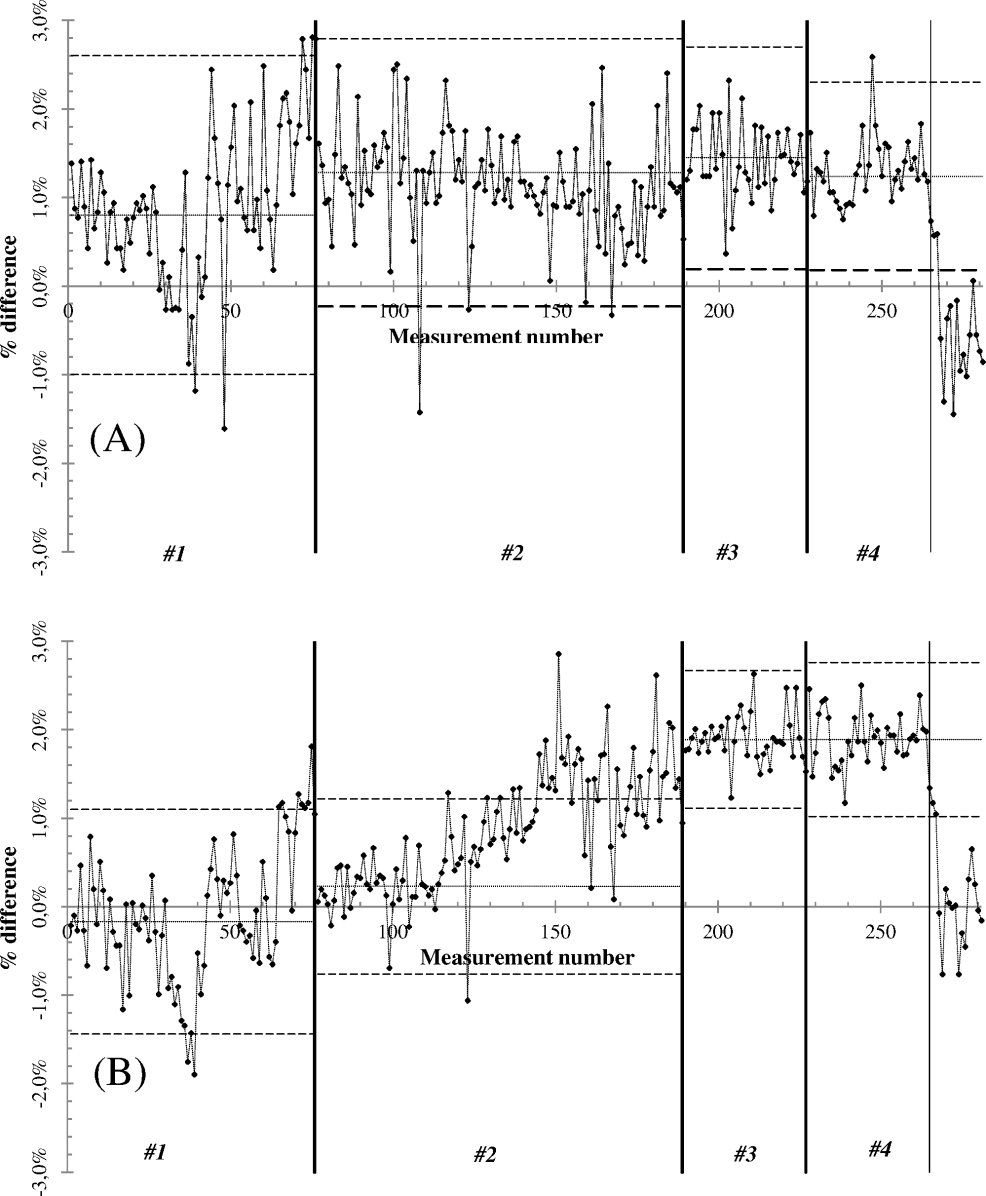
Individual value X-chart for the A) static and B) dynamic output checks during the observation period. The four datasets (#1, #2, #3 and #4) refer to different ionization chambers used during the observation period.

During the acquisition period, daily static and dynamic output data where within ±3% of their reference values. Individual X-charts for the 281 data points are shown in Fig 2. The whole dataset was divided into four groups on the basis of the ionization chamber used at the time. In both graphs of Fig 2A and 2B, the vertical bold solid lines represent the end of each group, the horizontal dotted lines represent the UCL and LCL, and the horizontal dashed lines represent the CL of each group.

**Fig 3:**
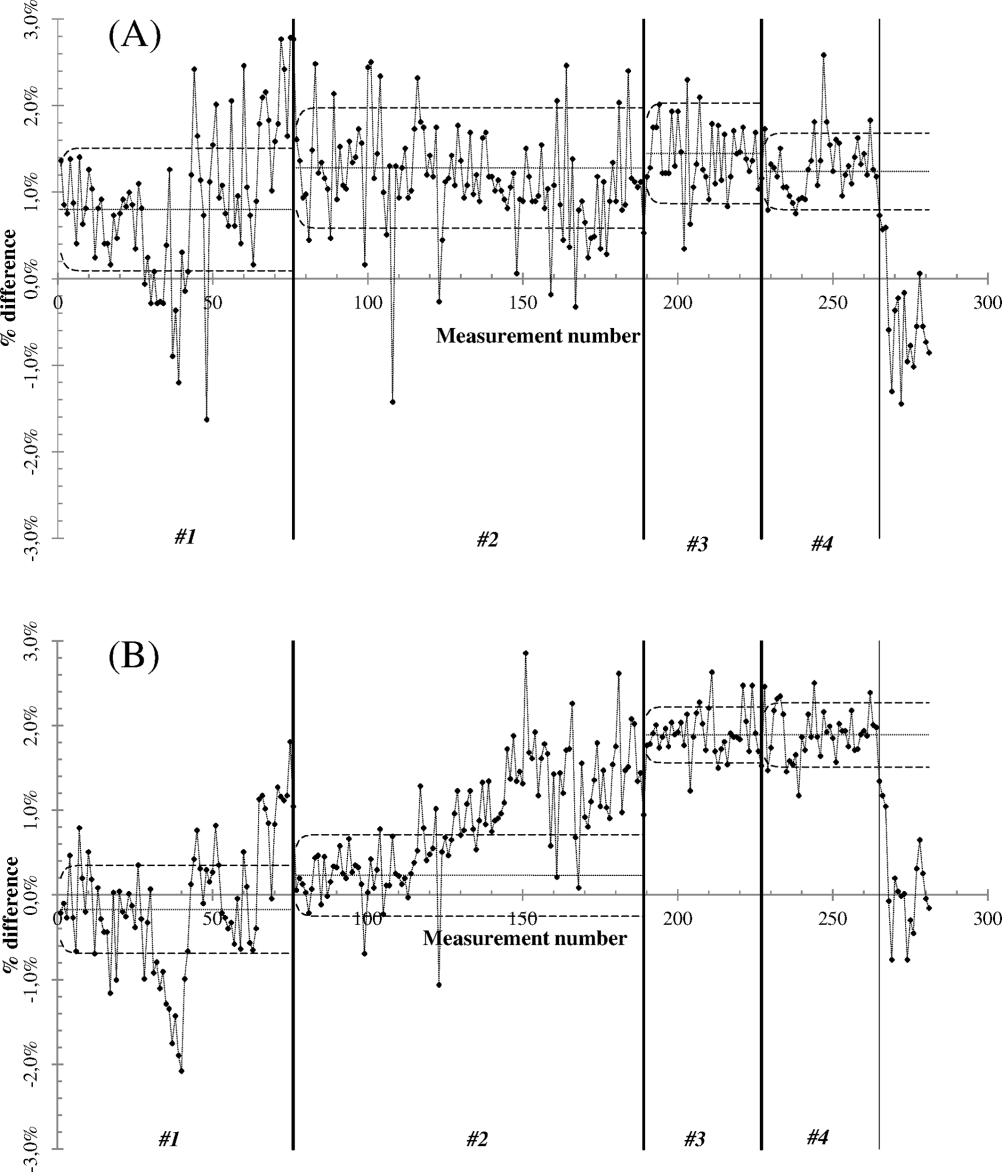
A) Static and B) dynamic output EWMA charts. The datasets (#1, #2, #3 and #4) correspond to the same datasets shown in Fig 2.

The EWMA charts (Fig 3A for static and Fig 3B for dynamic) were divided into four groups (corresponding to the same nomenclature adopted in Fig 2) to monitor variability in the process mean for the different ionization chambers used. The X and EWMA chart computed parameters are reported in Table 1, together with the X-chart acceptance window (defined as the difference between UCL and LCL).

**Table 1:** SPC parameters for the static and dynamic outputs.

Ion chamber	Output check	AD* ^a^ (99% critical, value = 1,092)	Probability test results	No. Measurements	X-chart ^b^	Acceptance window (%)	EWMA chart (%) ^c^
#1	Static	0.565	Normal	76	-1.00/0.80/2.60	3.60	0.29/0.80/1.31
	Dynamic	0.318	Normal	76	-1.44/-0.17/1.10	2.54	-0.55/-0.17/0.21
#2	Static	0.708	Normal	113	-0.23/1.28/2.79	3.02	0.78/1.28/1.78
	Dynamic	1.682	Not normal	113	-0.76/0.23/1.22	1.98	-0.11/0.23/0.57
#3	Static	0.501	Normal	38	0.19/1.45/2.70	2.51	-1.03/1.45/1.87
	Dynamic	0.683	Normal	38	1.11/1.89/2.67	1.56	1.65/1.89/2.13
#4	Static	19.95	Not normal	54	0.18/1.24/2.30	2.12	0.92/1.24/1.56
	Dynamic	19.92	Not normal	54	1.02/1.89/2.76	1.74	1.62/1.89/2.16

^a^ AD*, Anderson-Darling test for normal data distribution.

^b^ The values are reported in the sequence: LCL/CL/UCL.

^c^ The values are reported in the sequence: LCL/CL/UCL.

For both static (Fig 2A) and dynamic (Fig 2B) output checks, all data were normally distributed at the 99% critical value, with the exception of group #2 in Fig 2B (dynamic output) and group #4 in Fig 2A and 2B (both static and dynamic outputs). These results are shown in Table 1 in the column referring to the Anderson-Darling test (AD* column), where group #4 values reported are above the 99% critical value of 1.092. Although it is sometimes argued that a precondition to the use of control charts is that data must be normally distributed, this is not strictly true [21]. Control charts are robust for normal distribution data, but during the first phase of their use the process may not be stable and hence data may not be normally distributed. In particular, the assumption of normally distributed data is unlikely in the retrospective analysis of a dataset where the aim is to analyze whether special causes responsible for out of control processes that lead to non normal data distribution are present.

The data of dataset #4 in Fig 2A and 2B are not normally distributed. In fact, the vertical dotted line refers to the magnetron break and its subsequent replacement in our HT system. The points reported in Fig 2A and 2B after the magnetron substitution were insufficient for further control limit computation but are shown for completeness. Our results on SPC parameters for static and dynamic outputs (Table 1), together with those of the X-chart plots, indicate that the control limits were smaller than the ±3% window proposed by the AAPM TG 148. Of note, the acceptance window width decreased in datasets #1–4, showing an acceptance level lower than ±3%. Although the acceptance window width decreased, the CL of each dataset tended to increase, becoming fairly stable between the #3 and #4 datasets for both static and dynamic output checks. The differences in the central values of the corresponding groups in Fig 2A and 2B are potentially ascribable to variations in delivery time for the static and dynamic outputs (leading to different readings in static and dynamic output measurements). The similar trend for both static and dynamic output indicates the proper functioning of the DCS in automatically adjusting the dose rate output of the HT system.

For dataset #1 in Fig 2A and 2B, measurement no. 48 was present in the static (Fig 2A) but not dynamic output (Fig 2B). This was due to the incorrect positioning of the ionization chamber inside the phantom, as reported by the operator on that particular day. This situation is shown in the static EWMA chart (Fig 3A) at the same measurement number in which an abrupt displacement of the data trend was reported. In the EWMA chart (Fig 3A and 3B, dataset #1) three and two groups of out of control data were detected for static and dynamic outputs, respectively. For the static output check, the first group was a drift that was not detected in the X-chart; it did not correspond to a situation of out of control data but rather represented a data drift towards a good agreement with the static reference value (within a 0.2% difference for the 7 points around measurement no. 30 in Fig 2A). The second group of out of control data corresponded to an out of control group for which no assignable causes were detected (measurement no. 39). For the dynamic output check, Fig 3B shows a group with an out of control maximum that corresponds to measurement no. 39. The maximum value reached by the previous 8 measurements was the result of bunker pressure and temperature values higher than their reference values. As soon as the environmental conditions in the HT bunker were restored (after measurement no. 39), the static and dynamic output checks increased and oscillated around their CL value until the end of the dataset, where both outputs moved towards the UCL (reported in the EWMA charts of Fig 3A and 3B of the group of 10 data points out of control, dataset #1). From the maintenance report of the HT system, it was found that an incorrect jaw positioning occurred soon after measurement no. 75, requiring a field specialist intervention on the HT system.

The process dispersion was different for dataset #2 in Fig 2A and 2B, as clearly shown. Although the static output was within the control limits (as confirmed in the EWMA chart, Fig 3A) with the exception of data point 108 reported as a non correct chamber positioning inside the phantom, the dynamic output revealed an increasing trend starting from measurement no. 119 until measurement no. 156, after which the dynamic output checks oscillated around the UCL. This situation was confirmed in the EWMA chart (Fig 3A) where an increasing trend of the dataset was found starting from measurement no. 123 in the plot. In the same measurement range and continuing to measurement no. 175, the static output showed a decreasing trend with respect to the dynamic output check, after which it began to oscillate around its CL. Examining the maintenance reports and consulting the field specialist engineer of our HT system, a DCS failure was expected and preventive maintenance was carried out. Measurement nos. 118, 123, 151 and 154 were associated with the reported HT error (Fig 2A and 2B). The following out of control points which had assignable causes in dynamic output checks were points 145 and 147 (multi leaf collimator error), and 99 and 139 (water flow fault).

**Fig 4:**
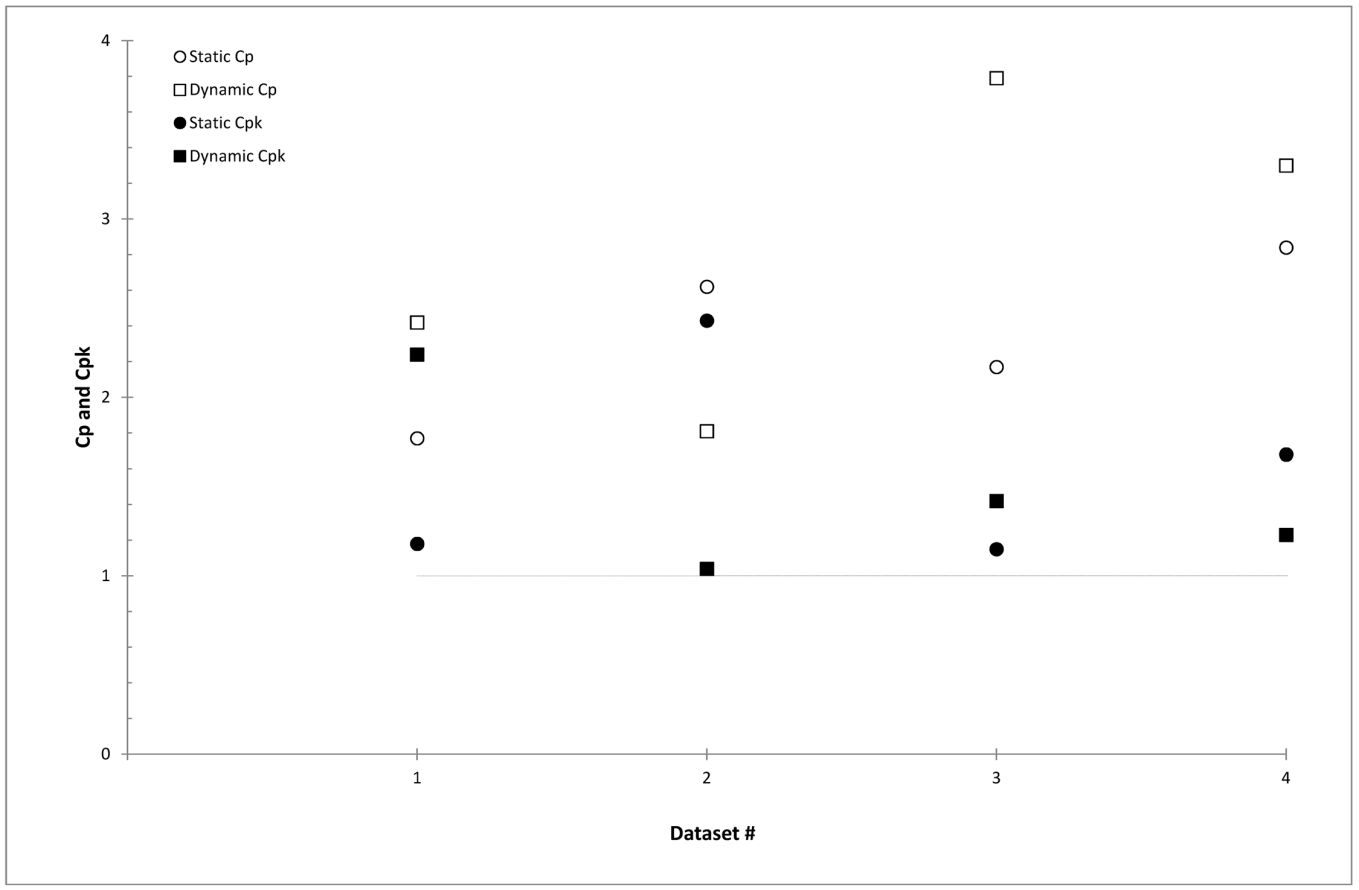
The capability (C_p_) and acceptability (C_pk_) index for static and dynamic output checks considered in Fig 2. The numbers #1, #2, #3 and #4 refer to the same dataset shown in Fig 2. C_p_ and C_pk_ values above the horizontal dashed line were considered acceptable.

Fig 4 shows a comparison between the C_p_ and C_pk_ obtained for each dataset in Fig 2. Their values are reported in Table 2. C_pk_ values were lower than C_p_ values for all datasets, which implies that the process shifted away from the target values, as seen in the X-charts.

**Table 2:** Process index values for process capability (C_p_) and acceptability (C_pk_).

Ion chamber	Output check	C_p_	C_pk_	C_pu_^a^	C_pl_^b^	P(%) ^c^	s
#1	Static	1.62	1.18	2.04	1.18	61.73	0.62
	Dynamic	2.37	2.24	2.24	2.51	42.19	0.42
#2	Static	2.62	2.43	2.83	2.43	38.17	0.38
	Dynamic	1.81	1.04	2.59	1.04	55.25	0.55
#3	Static	2.21	1.15	3.30	1.15	45.25	0.45
	Dynamic	3.84	1.42	6.27	1.42	26.04	0.26
#4	Static	2.88	1.68	4.04	1.68	34.72	0.35
	Dynamic	3.34	1.23	5.43	1.23	29.94	0.30

^a,^ C_pU_ are calculated as: C_pU_ = (USL-$\bar{x}$)/3s and: C_pL_ = ($\bar{x}$-LSL)/3s.

^b^ C_pL_ are calculated as: C_pU_ = (USL-$\bar{x}$)/3s and: C_pL_ = ($\bar{x}$-LSL)/3s.

^c^ P (%) is calculated as: P (%) = (1/C_p_)*100.

Based on the data presented in Table 2, the process was within the specification limit for the first dataset of Fig 2B (dynamic output check), but towards the lower specification (C_pk_ = 2.23). In Fig 2B, the CL of the process was slightly below (-0.17%) the target value, in agreement with the C_pk_ value. Conversely, for the other datasets of Fig 2A and 2B, the C_pk_ value indicated a process towards the upper specification limit, requiring corrective actions to the HT system. For the static output check shown in Fig 2A (datasets #1 and #4), the C_pk_ values were in agreement with the non-normality distribution of the same data. Their C_pk_ values near 1.00 indicate that in the observed process a situation whereby the output checks could not be considered acceptable was created. The dynamic output of this dataset showed an increasing trend (Fig 2B, dataset #2), while a magnetron replacement took place in dataset #4 (Fig 2A and 2B). In terms of the percentage of specification used [21] in the HT output checks (Table 2), it is worthy of note that, whilst the static and dynamic outputs were clinically acceptable [9], there was a continuously decreasing trend in the percentage of points within the control limits. This was observed for both static and dynamic output checks.

## Conclusion

This preliminary retrospective work investigated the usefulness of SPC methods applied to a HT system in detecting out of control situations with regard to static and dynamic output checks. Our results highlighted that a thorough and detailed analysis of various components of the HT system should be made during the real-time analysis of the output check process. We are thus planning to focus on some HT parameters (*i*.*e*. starting injector current, DCS operating window, ionization chamber offset from the beam isocentre, and maintenance intervention on the HT system) to perform a detailed static and dynamic output check analysis with the SPC method used in the present study. Our research will take into account different process control rules to identify situations that could be potentially correlated with the HT system state. This would help to optimize HT system maintenance interventions and facilitate corrective actions on the system itself.

## Abbreviations

QC: quality control
SPC: Statistical Process Control
CL: center line
UCL: upper control limit
LCL: lower control limit
HT: helical tomotherapy
DCS: dose control system
TPS: treatment planning system
IMRT: Intensity Modulated Radiation Therapy
EWMA: exponentially weighted moving range
H_0_: null hypothesis
AD: Anderson-Darling
C_p_: capability ratio
C_pk_: acceptability ratio
